# Understanding Fibroblast Heterogeneity in Form and Function

**DOI:** 10.3390/biomedicines11082264

**Published:** 2023-08-14

**Authors:** Jennifer B. Parker, Caleb Valencia, Deena Akras, Sarah E. DiIorio, Michelle F. Griffin, Michael T. Longaker, Derrick C. Wan

**Affiliations:** 1 Division of Plastic and Reconstructive Surgery, Department of Surgery, Stanford University School of Medicine, Stanford, CA 94305, USAmgriff12@stanford.edu (M.F.G.); 2 Institute for Stem Cell Biology and Regenerative Medicine, Stanford University School of Medicine, Stanford, CA 94305, USA

**Keywords:** fibroblast, fibrosis, heterogeneity, dermis, wound healing

## Abstract

Historically believed to be a homogeneous cell type that is often overlooked, fibroblasts are more and more understood to be heterogeneous in nature. Though the mechanisms behind how fibroblasts participate in homeostasis and pathology are just beginning to be understood, these cells are believed to be highly dynamic and play key roles in fibrosis and remodeling. Focusing primarily on fibroblasts within the skin and during wound healing, we describe the field’s current understanding of fibroblast heterogeneity in form and function. From differences due to embryonic origins to anatomical variations, we explore the diverse contributions that fibroblasts have in fibrosis and plasticity. Following this, we describe molecular techniques used in the field to provide deeper insights into subpopulations of fibroblasts and their varied roles in complex processes such as wound healing. Limitations to current work are also discussed, with a focus on future directions that investigators are recommended to take in order to gain a deeper understanding of fibroblast biology and to develop potential targets for translational applications in a clinical setting.

## 1. Introduction

With roles in growth and development, injury response, and tissue homeostasis, fibroblast functions within the body are dynamic and multifarious [1,2]. Their diversity in form and function has highlighted the striking heterogeneous nature of these cells. Not only that, fibroblasts are also known to exhibit substantial plasticity, which enables them to participate in a wide range of cellular and molecular activities [3,4,5].

Though present in all connective tissue, significant strides have been made in the study of fibroblasts within the skin [6,7,8,9,10,11,12,13]. As the primary barrier to the external environment, the ability of this organ to be able to repair itself swiftly so as to avoid additional damage or infection is crucial to animal survival. Oftentimes, it is the physical barrier protecting the body from the external environment that requires repair, which the body elegantly performs through a complex and multistage process of wound healing [14]. During wound repair, fibroblasts are one of the major cell types involved in the signaling and direct closure of the wound [15].

A skin scar is one of many examples of fibrotic tissue found within the body, wherein healthy native tissue is replaced by a dense connective tissue that often leads to a reduction in function [16]. As fibroblasts are a primary cell type in the development of fibrotic tissue, significant work is being performed to investigate the heterogeneous nature of fibroblasts alongside their diverse functions [17]. In this review, we will use the lens of wound healing to discuss the diverse anatomical and embryonic localization of fibroblasts along with the specific functions that they play.

## 2. Wound Healing

In response to injury, the skin undergoes a series of complex processes to achieve wound repair. The stages of wound healing include (1) hemostasis, (2) inflammation, (3) proliferation, and (4) remodeling/maturation. Within each phase, fibroblasts play an important role.

Wound healing begins with hemostasis, which is characterized by platelet aggregation at the site of injury to prevent blood loss [18]. The clotting cascade leads to the formation of a fibrin clot, eventually mobilizing immune cells to the wound site to contribute to further proinflammatory cytokine production and sterilize the affected area [18]. Macrophages in particular eliminate pathogenic material and modulate the shift from an inflammatory to regenerative phenotype [19,20].

The inflammatory reaction recruits fibroblasts to the wound site to deposit new ECM and re-establish the tissue architecture [21]. Now in the proliferation stage of wound healing, mechanical forces at the wound bed and several cytokines such as transforming growth factor-β1 (TGF-β1) drive fibroblast-to-myofibroblast differentiation [22]. These are modified fibroblasts containing smooth-muscle-like features with contractile properties [23]. Activated myofibroblasts are responsible for wound contracture and contribute to the development of collagen-rich ECM [24]. In postnatal wound healing, significant activation of fibroblasts following the inflammation stage typically leads to excessive and disorganized matrix deposition, ultimately giving rise to fibrosis or scarring [25,26].

The remodeling stage begins 2 to 3 weeks following initial injury, and may last over a year [15]. At this stage, many of the cells involved in the inflammatory and proliferative phases leave the wound or undergo apoptosis, leaving a cell-poor and collagen-rich aggregate [15]. In the latter half of the remodeling phase, this acellular matrix undergoes active regeneration and continuous turnover such that its primary composition changes from type III to type I collagen [27]. Over time, the ECM stiffens and strengthens, eventually resulting in a mature scar [28].

## 3. Definition of Fibroblast

Fibroblasts were first identified in the 1800s by Virchow as “spindle-shaped cells of the connective tissue” [29]. In 1895, Ernst Ziegler termed these cells as “fibroblast” [30]. Today, fibroblasts are defined in a number of different ways. Morphologically, fibroblasts typically appear spindle-shaped and elongated, and when activated and differentiate into myofibroblasts, they can spread and become stellate in appearance [23]. Though there are some markers considered typical of fibroblasts, such as vimentin and collagen, these are considered nonspecific [2]. PDGFRA is often used as a ‘lineage marker’; however, it has been shown that some fibroblasts do not express PDGFRA. Instead of identifying fibroblasts based on marker, an alternative approach used is to select fibroblast lineage by exclusion, by identifying cells that do not express markers known to be specific to other cell types, such as CD45 for lymphocytes and PECAM1 for vascular cells [31,32]. That said, by using a lineage negative approach, researchers may not be excluding all cells that are not fibroblasts. Overall, a persistent challenge researchers face is thus the study of fibroblasts in the absence of a unified marker.

In homeostasis, fibroblasts are responsible for depositing and maintaining collagen and proteins of the extracellular matrix (ECM), and as previously mentioned, in wound healing, fibroblasts contribute to the formation of new connective tissues by proliferating and secreting collagen [27,33]. These cells are also known to play roles in cancer, angiogenesis, and inflammation [17]. In cancer, for example, studies have demonstrated that fibroblasts secrete growth factors that promote tumor cell proliferation [34]. Fibroblasts are also known to secrete cytokines such as Interleukin 8 (IL-8), which are important in inflammation [35]. One of the challenges that researchers face in the study of fibroblasts is the absence of a unified marker.

While it was previously believed that fibroblasts are a homogeneous cell type, fibroblasts exhibit remarkable heterogeneity and plasticity, with distinct subtypes of fibroblasts involved in different biological processes [36]. The advances in single-cell analysis provide a different approach to classifying fibroblasts. Single-cell RNA sequencing (scRNA-seq) has allowed scientists to group cells based on the expression of multiple markers. This technique utilizes mRNA expression within cells to examine the gene expression profiles of individual cells, which can subsequently be used for differential gene expression analysis between and within cell types [37]. Today, papillary (CD26^+^SCA1^−^) and reticular (DLK1^+^SCA1^−^) fibroblasts are well-established subpopulations with differing gene expression and properties [38]. A recent scRNA-seq study of human dermal fibroblasts revealed that around 90% of dermal fibroblasts can be organized into three distinct groups. The groups are organized based on the expression of genes such as *SFRP2*, *APOE*, and *SFRP1*. These three distinct groups are further separated into 10 subgroups [39]. The advances in lineage tracing and single-cell analysis have thus provided evidence that supports the hypothesis that fibroblasts are heterogeneous in terms of function and phenotype [1].

## 4. Embryonic Origins

The embryonic origin of fibroblasts has been found to play a significant role in their heterogenic properties. Cell fate-mapping analysis has revealed that the embryonic origins of fibroblasts heavily depends on their anatomical location within the body [40]. Therefore, not all fibroblasts are derived from the same embryonic origin. Facial and dorsal fibroblasts contain different embryonic lineages and consequently have different scarring properties. Facial fibroblasts are known to arise from the neural crest, while dorsal fibroblasts are derived from the dermato-myotome, and ventral fibroblasts from the lateral plate mesoderm [1].

To better understand fibroblasts, it is important to determine whether a certain property, like scar formation, is an intrinsic or extrinsic characteristic. In a reciprocal transplant study, cells from the oral and dorsal dermis were transplanted to their respective counterparts [41]. Normally, scar formation is absent in the oral cavity; however, upon wounding, dorsal fibroblasts transplanted to the oral cavity led to scar formation [42]. This experiment suggested that scar formation is an intrinsic characteristic of fibroblasts [41].

**Figure 1:**
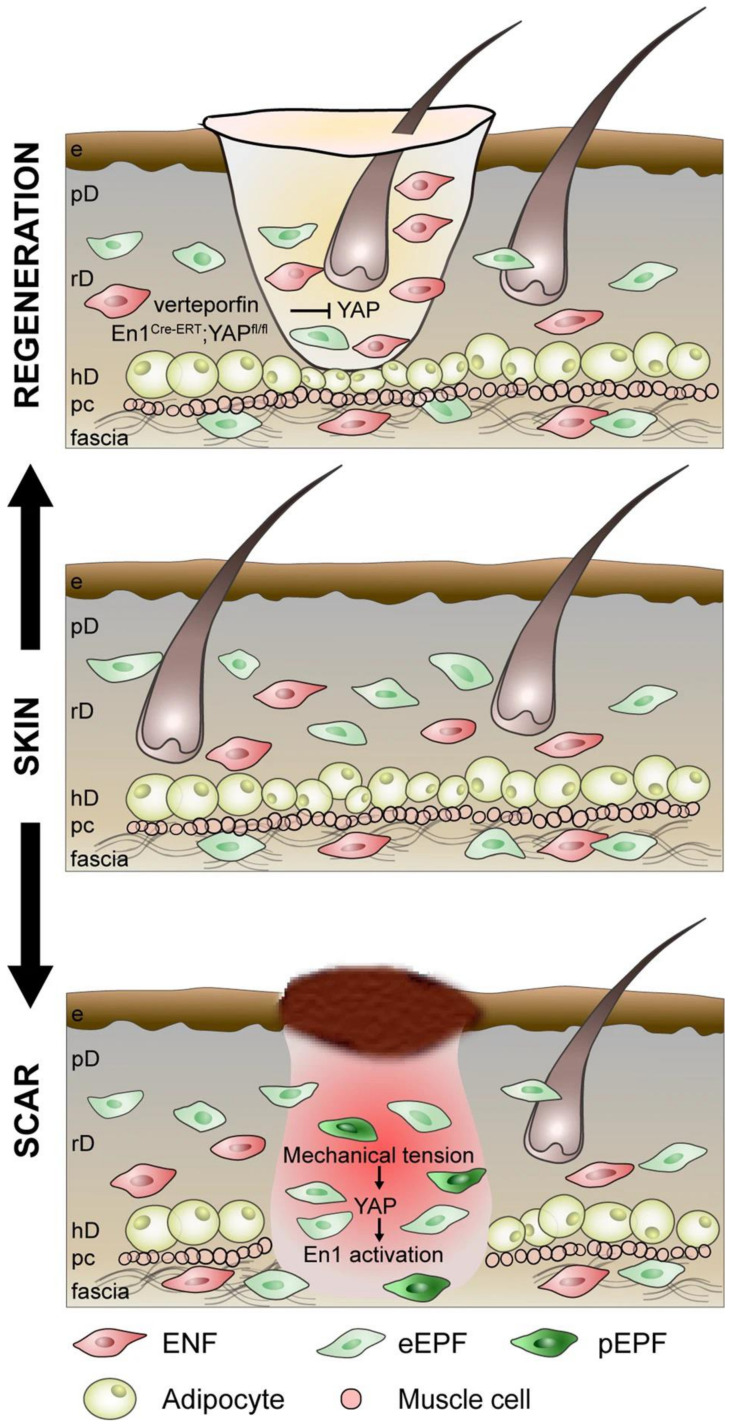
*Engrailed*-1 positive fibroblasts are the key fibroblast subpopulation contributing to dermal scarring in the mouse dorsum. During injury, a subpopulation of fibroblasts, *Engrailed-1* positive fibroblasts (EPFs) are the primary fibroblast contributing to skin scarring. Due to mechanical tension within the wound bed, Yes-Associated Protein (YAP) signaling activates *Engrailed-1* and subsequent scar deposition. Inhibition of YAP signaling chemically via verteporfin or with the use of a genetic knock-out prevents activation of *Engrailed-1*, leading to reduced scar deposition and a regenerative phenotype. Figure taken with permission from “Converting fibroblastic fates leads to wound healing without scar” by Jiang and Rinkevich [43].

Markers to distinguish different embryonic lineages have been identified. *PDGFRA*, delta-like homolog 1 (*DLK1*), and leucine-rich repeat protein (*LRIG1*) are a set of markers used to label multipotent mesenchymal cells with the ability to differentiate into all types of dermal fibroblasts [1]. Our laboratory recently identified four distinct embryonic fibroblast lineages: *Engrailed-1* positive and negative fibroblasts (EPFs and ENFs) and *paired-related homeobox 1* (*Prrx1*) positive and negative fibroblasts (PPFs and PNFs) [41]. EPFs have been found to play a significant role in connective tissue deposition during wound healing (Figure 1). In fact, scarring can be prevented when inhibiting EPF activation in dorsal wounds [9]. PPFs are responsible for the formation of scars on the ventral dermis of the mouse and deposit the majority of collagen during wounding. Correspondingly, the removal of PPFs resulted in decreased scarring [8].

## 5. Anatomical Variation

Though found in almost every tissue in the body, fibroblasts differ in phenotype and function across organs. As alluded to, this variation is largely attributed to distinct lineages of fibroblasts [1]. Those that contribute to tissue regeneration such as in the intestine assist in maintaining the stem cell niche and promote cell proliferation [44,45]. Meanwhile, in tissues such as the lung and liver where regeneration is only activated following injury, fibroblasts primarily work to restore the wound site and deposit ECM [46,47].

Fibroblast heterogeneity can be attributed to anatomical site in addition to developmental origin. On a whole-body scale, fibroblast gene expression has been related to position in terms of the anatomical axes (e.g., anterior vs. posterior) [48]. Additionally, fibroblast gene expression is linked to homeobox (HOX) gene expression and site of origin [49]. In one study, fibroblast cultures were created from adult skin derived from ten different anatomical sites (back, arm, abdomen, scalp, foreskin, thigh, gum, toe), and transcriptional analyses were performed. These investigations highlighted that the anatomical site of origin and fibroblast gene expression were strongly correlated [49]. In another study, fibroblasts demonstrated retained memory of their native niche when transplanted to a different part of the body, maintaining their original phenotype [50]. For instance, scalp dermis-derived fibroblasts transplanted into the arm support the creation of long hairs, indicating that these cells retain some intrinsic memory of their initial anatomical site even in the absence of the native niche. 

Fibroblasts that share an embryonic origin display heterogeneity based on their location in the body and the surrounding microenvironment [48]. The skin itself exhibits substantial diversity. The dermal layer, consisting of the papillary dermis, reticular dermis, and hypodermis, contains specialized fibroblasts that carry out different functions and arise from varying lineages. The erector pili muscle and dermal papilla fibroblasts, responsible for piloerection and the formation of hair follicles, respectively, share a common origin with those of the papillary dermis. Similarly, adipocytes, adipocyte progenitor cells, and reticular fibroblasts share common lineages, yet exhibit varying phenotypes [6]. Papillary and reticular fibroblasts both arise from dermal fibroblast progenitors that express *Pdgfrα*, *twist-related protein 2* (*Twist2*/*Dermo1*), and *En1* [45]. Papillary fibroblasts have been shown to distinctly express *Fap*, while reticular fibroblasts express *CD90* [51]. Evidence suggests that compared to reticular fibroblasts, papillary fibroblasts can be important players in scarless regeneration [52].

As alluded to, in addition to diversity in form, fibroblast subpopulations exhibit variation in function as well, which will be elaborated on in the following section.

## 6. Heterogeneity in Function

### 6.1. Fibrosis

Within the dermis, fibroblasts contribute to fibrosis differently based on their subtype. As mentioned, in dorsal skin, for example, EPFs are key contributors to scar formation, while PPFs are responsible for fibrosis following injury in the ventrum [8,9]. In contrast, their counterpart populations, *En1* and *Prrx1* lineage-negative fibroblasts, have displayed nonfibrogenic properties and enable proregenerative healing [9,41]. Notably, behaviors of these subpopulations are intrinsic to the cells. When EPFs or PPFs are transplanted into oral mucosa, which normally does not scar, the fibrogenic properties of these cells persist [8,9].

Wound healing also illustrates the breadth of contribution fibroblast subpopulations have to fibrosis. Smaller wounds, for instance, show increased involvement of reticular and hypodermal lineages, whereas larger wounds involve papillary fibroblasts, and a subset of reticular fibroblasts involved in adipocyte regeneration [6]. Further, the embryonic origins of fibroblast subpopulations, described in a previous section, may also affect the extent of fibrotic behavior [53], with EPFs and PPFs derived from the paraxial and lateral plate mesoderm, respectively, contributing to scarring in the trunk. In contrast, *Wnt1*-lineage-postitive fibroblasts, derived from the neural crest, allow for the oral mucosa to heal without a scar [41,54].

Although the body has the remarkable ability to heal dermal wounds, there are multiple factors that may lead phases of wound healing to go awry. For instance, chronic wounds fail to close, and include wounds such as diabetic foot ulcers, pressure ulcers, and venous leg ulcers [55]. Keloid scars continue depositing fibrotic scar tissue, presenting as nodules in areas of prior injury often extending far beyond the initial site of trauma [56]. Scleroderma is a disease characterized by cutaneous sclerosis, or hardening [57]. Fibroblasts are essential players in pathological fibrosis, involving conditions such as hypertrophic scars, keloids, and systemic sclerosis [58]. During the process of pathological scar formation, fibroblasts undergo increased proliferation and secrete excessive collagen, leading to scar formation [6]. Moreover, in the context of chronic wounds, *En1*-positive fibroblasts are identified as the primary cells responsible for depositing collagen [41]. Fibroblasts are believed to be the main culprit when it comes to systemic sclerosis, replacing normal dermis with dense, stiff connective tissue. The tightness of the skin could be attributed to an increased deposition of the matrix, which is closely relate to the presence of myofibroblast in the skin [59].

### 6.2. Plasticity

On top of the different subpopulations of fibroblasts, and the different roles these fibroblasts play as a result, their heterogeneity is also illustrated by the ability of these cells to interconvert with other cell types. In the context of scarring, lung and skin fibroses show transition of adipocytes, fascial fibroblasts, pericytes, and hematopoietic cells into myofibroblasts [12,60,61,62].

A broad notion relevant to fibroblast heterogeneity revolves around epithelial-to-mesenchymal transition (EMT). There are two forms of EMT. Type I refers to EMT that occurs during development, such as the transition of epicardial epithelial cells to cardiac fibroblasts in the developing heart [63]. In contrast, type II EMT occurs into adulthood in instances of injury. For example, in response to injury, epithelial-to-myofibroblast transition has been shown in renal, liver, and pulmonary fibrosis. Though transdifferentiation of epithelial cells to fibroblasts may represent a particular subpopulation of myofibroblasts, the mechanistic pathways underlying EMT activation remain elusive [64].

Another form of transition that has been described in other forms of fibrosis, including subretinal and renal fibroses, is macrophage-to-myofibroblast transition (MMT) [65,66]. In addition to the role immune cells play in secreting cytokines and chemokines that lead to fibroblast proliferation, bone-marrow-derived macrophages (BMDMs) have been shown to directly contribute to renal fibrosis through MMT. These cells secrete collagen and are distinguishable from other fibroblast subpopulations by their expression of CD68, a macrophage marker. A study investigating a unilateral ureteral obstruction model for renal fibrosis demonstrated that 35% of myofibroblasts originated from bone marrow cells [66]. Similarly, in the context of subretinal fibrosis, investigators determined that 20–30% of infiltrating F4/80+ macrophages in subretinal fibrotic lesions from human neovascular age-related macular degeneration eyes and murine subretinal fibrotic lesions co-expressed α-SMA [65]. Though they acknowledged that further research will be required to understand the extent of MMT’s role in macular fibrosis, the investigators concluded that MMT is a contributor to the pathology.

Cellular memory may also be an important variable in considering fibroblast plasticity. For example, history of prior injury may affect present wound healing biology. It has been demonstrated that epidermal stem cells (EpSCs) have inherent memory after an acute inflammatory event, with EpSCs retaining chromatin accessibility of stress-response genes for as long as 180 days following injury. Retained chromatin accessibility allowed for accelerated transcription of these same genes after a secondary insult, effectively enabling these EpSCs to be primed and respond more efficiently during instances of injury [67]. Genes associated with wound memory included inflammation, cell migration, and cytoskeletal reorganization [68]. In an organ that consistently is required to respond to insults from the external environment, the ability for cells within the skin to retain cellular memory to respond more efficiently to injury could be the result of an evolutionary trait. Notably, a recent study demonstrated that cells distal to a wound site that originally were not activated during the initial wound healing become primed at a chromatin and transcriptional level to enhance wound repair [69]. In parallel, as fibroblasts are a prominent cell type in the dermis, it would be of significant interest to determine whether fibroblasts similarly carry epigenetic ‘memory’ to improve response to injury [68].

DNA methylation is believed to be an important contributor to cellular memory as DNA methylation profiles can alter the expression profiles within cells. Importantly, DNA methylation profiles can be retained within specific cells and their progeny, thus leading to cellular subpopulations characterized by different DNA methylation profiles [70]. In the context of lung fibrosis, research groups have identified up to 11 subpopulations of fibroblasts in healthy and diseased states. Liu et al. recently demonstrated that there are 125 differentially methylated CpGs in idiopathic pulmonary fibrosis (IPF) fibroblasts when compared to healthy lung fibroblasts, and that these differentially methylated regions were also associated with altered gene expression [71].

It is believed that many of the processes of wound healing are regulated by epigenetic mechanisms [28]. For one, fibroblast maturation is directed by a combination of methylation and histone modifications, with methylation controlling the expression of aSMA during myofibroblast differentiation, specifically [72]. Other studies have also shown that DNA methylation leads to upregulation of profibrotic TGF-ß1 signaling [73,74]. During wound healing, epigenetic modulators appear to alter temporally and spatially. For example, full-thickness wounds have been shown to exhibit decreased H3K27me expression specifically at the wound edges, whereas DNA hypermethylation across the entire wound bed is seen during the re-epithelialization phase [75]. That said, as variability remains in how subpopulations of fibroblasts are defined, how epigenetic regulation may affect these subtypes and the dermal fibroblast pathway as a whole remains elusive.

## 7. Molecular Tools

Developments in molecular techniques have provided opportunities for investigators to conduct in-depth investigations into fibroblast heterogeneity as well as skin biology and repair. These methods continue to provide increased insights at transcriptomic, epigenetic, and proteomic levels (Table 1).

### 7.1. Single-Cell RNA Sequencing

scRNA-seq is one of the most widely applied methods used to explore fibroblast heterogeneity. Analysis typically begins by clustering cells in the dataset based on gene expression using computational methods, and annotation of these cluster groups based on cluster gene signatures. That said, parameters used to identify these clusters are user- and method-specific, and thus, subjective. For instance, one study investigating dermal fibroblasts from human skin identified five distinct fibroblast subpopulations, while a more recent group identified three major fibroblast types and 10 subtypes [39,76]. Importantly, to what extent these populations identified also differed in function based on assigned type is not yet clear. Different methodologies used to analyze ‘omic’ datasets leads to variability in the downstream fibroblast subpopulations assigned. Furthermore, the extent of quality control performed on a given dataset can lead to substantial changes to the conclusions that one draws. For example, a reanalysis of the Reynolds et al. dataset [77], which consisted of over half a million sequenced human adult skin cells, illustrated that identified transcriptomic signatures may be biased and the result of cellular stress and hypoxic conditions as opposed to subclustering of fibroblasts due to ‘true’ gene expression variations [78]. This work illustrated the importance of short processing time that would avoid undue stress on cells prior to sequencing. Additional investigations focused on improving consistency in both tissue collection and processing as well as downstream transcriptomic analyses and cluster configuration, along with functional studies used to validate the findings identified through computational methods, will be important next steps to solidify conclusions drawn by different investigators.

### 7.2. Downstream Transcriptomic Analyses

Excitingly, over the past few years, the field has had the opportunity to further investigate crosstalk among cell types with the development of a scRNA-seq tool called CellChat [79]. This tool was validated by comparing cell–cell interactions during adult skin wound healing and embryonic skin development. With their tool, they highlighted how myeloid and endothelial cells interacted with fibroblasts, and how communication between these cells differed dramatically when comparing embryonic skin to adult wound repair. 

**Figure 2:**
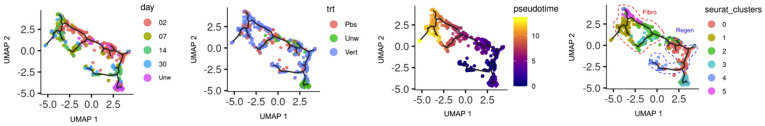
Pseudotime trajectory analysis as an example of computation tool to investigate fibroblast heterogeneity. Pseudotime trajectory analysis can be used to infer gene expression dynamics within a given group of cells. In this panel taken with permission from Mascharak et al. [10], differential gene expression between fibroblasts from control scars, from scars treated with an antifibrotic agent, and from unwounded skin, demonstrated a split into two major trajectories: fibrotic and regenerative.

One of the trade-offs of today’s transcriptomic techniques is that typical investigations will only provide a snapshot of a dynamic process. In the case of wound healing, one can sequence cells from different postoperative timepoints; however, the overlapping and continuous nature of wound healing may lead to differing interpretations due to the fixed timepoints selected. Tools have been developed in order to infer dynamics from static datasets. RNA velocity, for example, enables users to predict the future state of individual cells by analyzing spliced and unspliced mRNAs from scRNA-seq [80]. Pseudotime is another package used for inferred trajectory analysis (Figure 2) [81]. Though useful, the trajectory analyses still remain limited based on the number of timepoints and biological samples selected [82].

### 7.3. Spatial Techniques

A major drawback to most transcriptomic tools is the loss of spatial organization as a result of how cells are homogenized and processed for these techniques. As anatomical organization is vital to skin function, conserving spatial information may provide deeper insights into how spatial distribution of these cells within the tissue relates to heterogeneity in gene expression within fibroblast subpopulations. Novel methods preserving the spatial integrity of the tissue have opened up exciting avenues to investigate this relationship, including CODEX and Visium, which provide spatial proteomic and transcriptomic information, respectively. A few studies in the skin have applied these techniques to date, demonstrating that proteomic and transcriptomic fibroblast profiles group based on anatomical origin (epidermis, dermis, hypodermis), and that fibroblasts’ contribution to wound healing varies based on spatial organization [7,83,84].

## 8. Fibroblast Heterogeneity in Other Organ Systems

**Figure 3:**
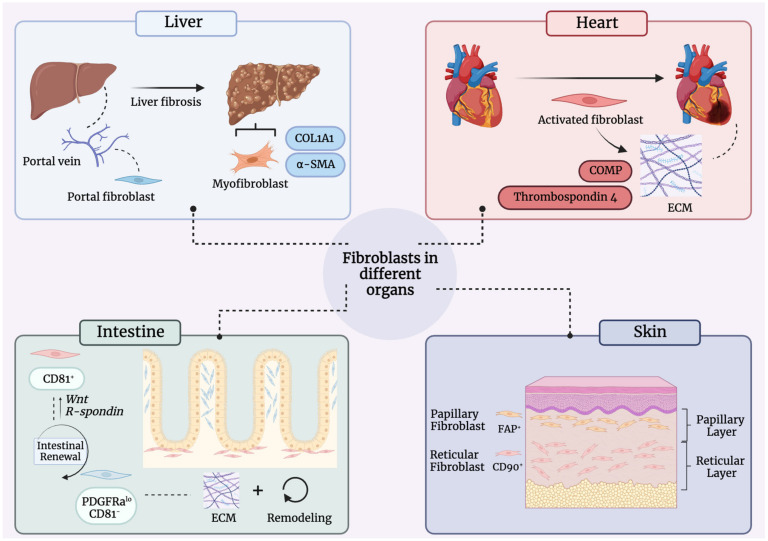
Fibroblast heterogeneity across organ systems. Fibroblasts exhibit differences in gene expression and function based on organ system. Liver fibroblasts can be found alongside the portal tract, termed portal fibroblast. Myofibroblasts expressing alpha-Smooth Muscle Actin (α-SMA) and Collagen Type I (COL1A1) can be found in the scar. Portal fibroblasts can be found along the portal tract (**top left**). Upon injury to the heart, cardiac fibroblasts are activated and deposit excess extracellular matrix (ECM) that uniquely contains Cartilage oligomeric matrix protein (COMP) and thrombospondin 4, both of which confer strength to the new scar but result in a functionally deficient scar (**top right**). In the intestine, two broadly defined subpopulations of fibroblasts are found: CD81^+^ and Platelet-Derived Growth Factor Alpha (PDGFRa)^lo^, CD81^−^. CD81^+^ fibroblasts (red) can be found beneath the crypts, which play a role in intestinal renewal. PDGFRa^lo^, CD81^−^ fibroblasts reside within the lamina propria and contribute to the production of ECM and remodeling (**bottom left**). Within the skin, subpopulations of fibroblasts exist within different layers of the skin. Papillary fibroblasts (yellow) are FAP^+^ cells found in the papillary dermis and believed to contribute to hair growth regulation, piloerection control, and late-stage wound repair. Reticular fibroblasts (red) are CD90+ cells found in the reticular layer of the skin and participate in fibrillar ECM formation and contribute to the initial stages of wound repair (**bottom right**).

Similar to what is seen in the skin, fibroblasts present in other organ systems demonstrate significant heterogeneity, as summarized in this section (Figure 3).

### 8.1. Liver

In the liver, fibroblasts can be found alongside the portal tract, termed portal fibroblast. Although there is limited understanding of the direct function of portal fibroblasts, they give rise to myofibroblasts that contribute to biliary fibrosis [85]. Hepatic stellate cells (HSCs) have been a focal point of research in liver disease. In a healthy liver, these cells are tasked with storing and releasing vitamin A [86]. However, during liver fibrosis, quiescent HSCs undergo activation and transdifferentiate into myofibroblast [87]. Myofibroblasts become abundant in the liver during injury and are responsible for excess fibrotic extracellular matrix deposition. Upon scar formation, myofibroblasts can be found embedded in the scar, and it has been suggested that a decrease in fibrosis correlates with decreased myofibroblast presence. Currently, the origin of myofibroblasts in the liver is unknown [88].

Myofibroblasts can be identified through two markers: alpha smooth muscle actin (*α-SMA*) and collagen type 1 (*COL1A1*) [89]. Morphologically, myofibroblasts are large spindle-shaped cells, which contain an active endoplasmic reticulum, a collagen-producing Golgi apparatus, and gap junctions [90].

### 8.2. Heart

Cardiac fibroblasts sit interspersed between cardiomyocytes and make up one of the heart’s largest cellular populations [91]. They arise from epicardial and endocardial epithelial cells during development through epithelial- and endothelial-to-mesenchymal transition (EMT and EndMT) [33]. This is in contrast to the fibroblasts of other organs that arise from progressive differentiation of mesenchymal progenitors [33].

Cardiac muscle is poorly regenerative. Following an injury such as myocardial infarction, cardiac fibroblasts activate proinflammatory and profibrotic signaling pathways and deposit excess ECM [92]. This ECM is unique in that it contains Cartilage oligomeric matrix protein (COMP) and thrombospondin 4, components normally found in bones, tendon, and cartilage [93,94]. These distinct factors are considered to confer strength to the new scar in order to withstand constant contraction of the heart in the future. Thus, myofibroblasts activated as a result of an infarction play a reparative role; if absent, the mammalian heart ventricle could risk catastrophic rupture [95].

The fibrotic response overall leads to the formation of a functionally deficient scar which can ultimately give rise to congestive heart failure and/or arrhythmias [15]. Developing treatments for cardiac fibrosis is complicated given its pathophysiologic heterogeneity [96]. Potential therapeutics being explored for the treatment of cardiac fibrosis include TGFβ1 and RAAS system inhibitors [97]. Cardiac lymphatics have also been shown to play an important role in cardiac fibrosis progression through mechanical stress [98].

### 8.3. Intestine

Significant work has been performed investigating the heterogeneity of fibroblasts within the intestine. In particular, distinct fibroblasts play key roles in intestinal homeostasis. For example, PDGFRa^lo^CD81^−^ fibroblasts residing around crypts, in the lamina propria, and within the villous core secrete basement membrane proteins and assist with ECM production and remodeling [99]. CD81^+^ fibroblasts, on the other hand, are found in the submucosa, proximal to vascular structures and below the crypts, and help maintain intestinal stem cell identity and proliferation [100].

Fibroblasts have also been shown to play a key role in the context of tissue damage and repair. Many studies have been performed analyzing the transcriptomic changes that occur during intestinal damage at a single cell level. All of these studies made use of a dextran-sulfate sodium (DSS) murine colitis model [101]. Some investigators suggest that the same subpopulations of fibroblast exist in the context of disease, but that their gene expression profiles are somewhat altered. For example, in the event of disease, inflammatory mediators, ECM components, and remodeling enzymes are all upregulated [102,103,104]. Further, single cell transcriptomic investigations indicate that some subtypes of fibroblasts play a crucial role in epithelial cell regulation during injury; CD81^+^ fibroblasts, for example, assist in intestinal repair through increased *Wnt* and *R-spondin* expression [105].

Lastly, various gene expression datasets have illustrated that all subsets of fibroblasts express proinflammatory genes in instances of intestinal damage or inflammation [101]. It is posited that elevation in proinflammatory genes leads to changes to immune cell recruitment and function. For example, in ulceration sites of human patients, IL-1ß specifically activates fibroblast-derived neutrophil-attracting factors [106]. Meanwhile, restricted activation of TNF signaling in fibroblasts alone is sufficient for intestinal pathology to develop [107].

### 8.4. Tendons and Ligaments

Tendons consist of a substantial population of fibroblast-like cells called tenocytes, distinguishable by their positive expression of Scleraxis (SCX) [108]. These tenocytes play a vital part in maintaining tendon homeostasis and facilitating repair through the production of collagen, fibronectin, and proteoglycans [109]. Notably, recent studies have revealed that the tenocyte population exhibit heterogeneity [110]. Ligaments also contain fibroblasts, which are termed ligamentocytes [111]. These cells bear resemblances to tenocytes in their capacity to produce collagen and contribute to the ECM [112]. Although our knowledge regarding ligamentocytes is somewhat restricted, some work has been conducted highlighting variations in collagen maturation among different ligaments, with fibroblasts derived from the anterior cruciate ligament (ACL), medical collateral ligament (MCL), and patellar tendon (PT) exhibiting different collagen maturation processes [113].

## 9. Future Directions

### 9.1. Characterizing Human-Specific Fibroblast Heterogeneity

As summarized here, significant strides have been made in characterizing fibroblast subpopulations in homeostatic and injured murine skin. Although some research has been conducted with human dermis, further investigations into markers that define subpopulations of human fibroblasts will be critical in order to gain an even deeper understanding of human homeostasis and disease [38,53]. This information would also provide insights into potential signatures for diagnosis and prognosis, as well as potential therapeutic targets.

### 9.2. Molecular Approaches and Current Limitations

In recent years, the significant strides made in molecular tools have enabled a much deeper investigation into skin biology and fibrosis at transcriptomic, epigenomic, and proteomic levels. scRNA-seq has permitted the identification of additional fibroblast subpopulation-defining markers, and when coupled with other computational tools such as CellChat, provides a wealth of information regarding associated signaling pathways and cell–cell interactions [79]. Challenges remain with the application of current single-cell transcriptomic approaches, including low cell capture efficiency, low sequencing resolution, and batch effects [82]. Notably, many of the parameters used for downstream analysis are subjective, and thus better establishing uniformity in applying these computational tools could assist in integrating data from different studies.

Given the three-dimensional nature of skin, and that fibroblast function differs based on anatomical location, developments of assays such as spatial transcriptomics (e.g., Visium) and CODEX have provided investigators with the opportunity to explore the additional dimension of spatial organization and how special organizing may affect fibroblast behavior during healthy and disease states [7,83,114]. Notably, though these tools provide a wealth of information, one of the current drawbacks with these spatial techniques lies in the fact that they can only visualize otherwise three-dimensional tissue in two dimensions. Future work applying three-dimensional analyses would therefore be an exciting addition to current molecular tools.

Most importantly, combining these different tools, and integrating transcriptional, epigenomic, and proteomic analyses together, allow for additional details to assist in defining fibroblast subpopulations and dynamics.

### 9.3. Translation

As suggested here, the study of fibroblasts has only begun to uncover the breadth of their profiles and functions; yet, no therapy specifically targeting scarring and promoting wound or organ repair and regeneration exists in a clinical setting to date [68]. Further explorations into the mechanisms that drive fibroblast dynamics during fibrosis using both small and large animal models, and how these mechanisms relate to human physiology and disease, could provide translational approaches for a sequela affecting millions of individuals across the globe.

## 10. Conclusions

Though previously believed to be connective tissue cells with little to no function, fibroblasts are increasingly believed to play crucial roles in both homeostasis and disease. In addition to their diversity based on embryonic and anatomic origin, these cells demonstrate substantial plasticity and breadth of function within fibroblast subtypes. Even with this improved understanding, many aspects of these cells need to be further explored, such as their plasticity in being able to transition into other cell types and better defining the true extent of their heterogeneity. Understanding how these cells, on a mechanistic level, can alter their function and influence cellular interactions with other cell types will be crucial to grasp a further holistic understanding of these intriguing cells. 

## Figures and Tables

**Table 1:** Molecular tools and the study of fibroblast heterogeneity.

Technique	Description
scRNA-seq	Used to investigate gene expression at a single-cell level. Allows researchers to explore variation in gene expression within a given cell type (e.g., fibroblasts).
Spatial Transcriptomics (e.g., Visium)	Gene expression in small groups of cells is measured and sequenced while retaining the spatial dimensionality of the tissue. Permits investigators to explore gene expression across cells and consider how gene expression may relate to overall tissue organization.
Spatial Proteomics (e.g., CODEX)	DNA-conjugated antibodies permit the visualization of up to 100 markers in situ, allowing one to study spatial relationships between individual cells at a protein level.
